# Pathophysiological Mechanisms and Potential Therapeutic Targets in Cerebral Autosomal Dominant Arteriopathy With Subcortical Infarcts and Leukoencephalopathy (CADASIL)

**DOI:** 10.3389/fphar.2020.00321

**Published:** 2020-03-13

**Authors:** Martina Locatelli, Alessandro Padovani, Alessandro Pezzini

**Affiliations:** Department of Clinical and Experimental Sciences, Neurology Clinic, University of Brescia, Brescia, Italy

**Keywords:** CADASIL, small-vessel disease, vascular cognitive impairment, stroke, ischemic, migraine with aura

## Abstract

Cerebral autosomal dominant arteriopathy with subcortical infarcts and leukoencephalopathy (CADASIL), is a hereditary small-vessels angiopathy caused by mutations in the NOTCH 3 gene, located on chromosome 19, usually affecting middle-ages adults, whose clinical manifestations include migraine with aura, recurrent strokes, mood disorders, and cognitive impairment leading to dementia and disability. In this review, we provide an overview of the current knowledge on the pathogenic mechanisms underlying the disease, focus on the corresponding therapeutic targets, and discuss the most promising treatment strategies currently under investigations. The hypothesis that CADASIL is an appropriate model to explore the pathogenesis of sporadic cerebral small vessel disease is also reviewed.

## Introduction

Cerebral autosomal dominant arteriopathy with subcortical infarcts and leukoencephalopathy (CADASIL), is a hereditary small-vessels angiopathy that usually affects middle-ages adults (Opherk et al., 2004). Typical clinical manifestations include migraine with aura, recurrent strokes, mood disorders, and cognitive impairment leading to dementia and disability (Chabriat et al., 2009). CADASIL is caused by mutations in the *NOTCH3* gene, involved in vascular smooth muscle cells maturation and homeostasis, located on chromosome 19 (Joutel et al., 1996). Despite the well-established etiology, uncertainties regarding pathogenesis have delayed development of effective treatment, and specific therapy is still unavailable.

In this review, we provide an overview of the current knowledge on the pathogenic mechanisms underlying the disease, focus on the corresponding therapeutic targets, and discuss the most promising treatment strategies currently under investigations. Whether an increased understanding of the pathophysiology and innovations in the therapeutic approach to CADASIL will also benefit patients with sporadic cerebral small-vessel disease (SVD) is also discussed.

### Epidemiology

With a prevalence of mutation carriers estimated between 0.8 to 5 per 100,000 individuals, CADASIL is considered, according to the European definition for rare disease (a disease affecting less than 1 person per 2,000), a rare disease (Razvi et al., 2005; Chabriat et al., 2009; Narayan et al., 2012; Moreton et al., 2014). However, recent data suggest a higher prevalence of *NOTCH3* pathogenic variants in the general population worldwide, with the highest frequency in Asiatic descendant, suggesting that CADASIL may manifest with milder clinical variants that currently remain undiagnosed (Rutten et al., 2016; Rutten et al., 2019). Accordingly, an extensive retrospective Italian study found a minimum prevalence of CADASIL of 4.1 per 100.000 adult inhabitants, significantly higher compared to that observed in two previous epidemiologic studies conducted in the northeast England and west of Scotland which reported a prevalence of 1.3 and 1.9 per 100.000, respectively (Razvi et al., 2005; Narayan et al., 2012; Bianchi et al., 2015). Similarly, as opposed to a median age at diagnosis which varies between 45–50 years, without substantial gender differences (Dichgans et al., 1998), Bianchi et al. found a higher mean age at diagnosis (57.8 years), with no differences in the latency from disease onset compared to other studies (from 3 to 14 years) (Bianchi et al., 2015). Despite huge variability in the clinical manifestations between subjects, all CADASIL patients inevitably progress until dementia and disability. More than half of CADASIL patients older than 58 years are unable to walk, and, by the age of 65, close to 65% are unable to attend their own bodily needs or require constant nursing care (modified Rankin Scale, 4–5) (Van Swieten et al., 1988; Dichgans et al., 1998; Moreton et al., 2014). Moreover, CADASIL reduces life expectancy, with mean age at death of 64,4 years in men and 70,7 years in women (Di Donato et al., 2017).

### Clinical Features

Although the clinical presentation may vary between patients, CADASIL is essentially characterized by four key symptoms: migraine with aura, recurrent ischemic strokes, psychiatric disturbances, and cognitive decline (Davous, 1998; Chabriat et al., 2009). Migraine with aura occurs in 20–40% of affected subjects and is usually the presenting symptom of the disease. Notably, one-half of patients complain of at least one atypical aura, and attacks frequency varies among individuals (Chabriat et al., 2009; Guey et al., 2016). Cerebral transient ischemic attacks and infarctions are the most frequent manifestations of the disease, occurring in 60–85% of symptomatic individuals (Chabriat et al., 2009; Drazyk et al., 2019). Age at onset ranges between 20 to 70 years and patients generally experience two to five ischemic events during lifetime, which over years result in motor and cognitive decline, gait disturbances, urinary incontinence, and pseudobulbar palsy (Chabriat et al., 2009; Opherk et al., 2004). Ischemic lesions are generally lacunar infarcts involving the subcortical white matter, clinically presenting as a classic lacunar syndrome. The second most frequent manifestation of CADASIL is cognitive impairment, occurring in 60% of patients. It becomes clinically detectable at 35–50 and progressively worsens with ageing and recurrent strokes, leading to alterations in verbal or visual memory, language, reasoning, and visuospatial abilities (Buffon et al., 2006).

Psychiatric disturbances occur in about 25–30% of patients, usually in the form of moderate/major depression, bipolar disorders, panic disorders, schizophrenia, and apathy (Lågas and Juvonen, 2001; Chabriat et al., 2009; Reyes et al., 2009). Less common clinical features of CADASIL include seizures, intracerebral hemorrhages, spinal cord signs, and parkinsonism (Baudrimont et al., 1993; Hutchinson et al., 1995; Choi et al., 2006). Although recent reports have suggested increased incidence of myocardial infarction, evidence of cardiac involvement in CADASIL is still conflicting (Lesnik Oberstein, S. A. J. et al., 2003).

### Diagnosis

**Figure 1:**
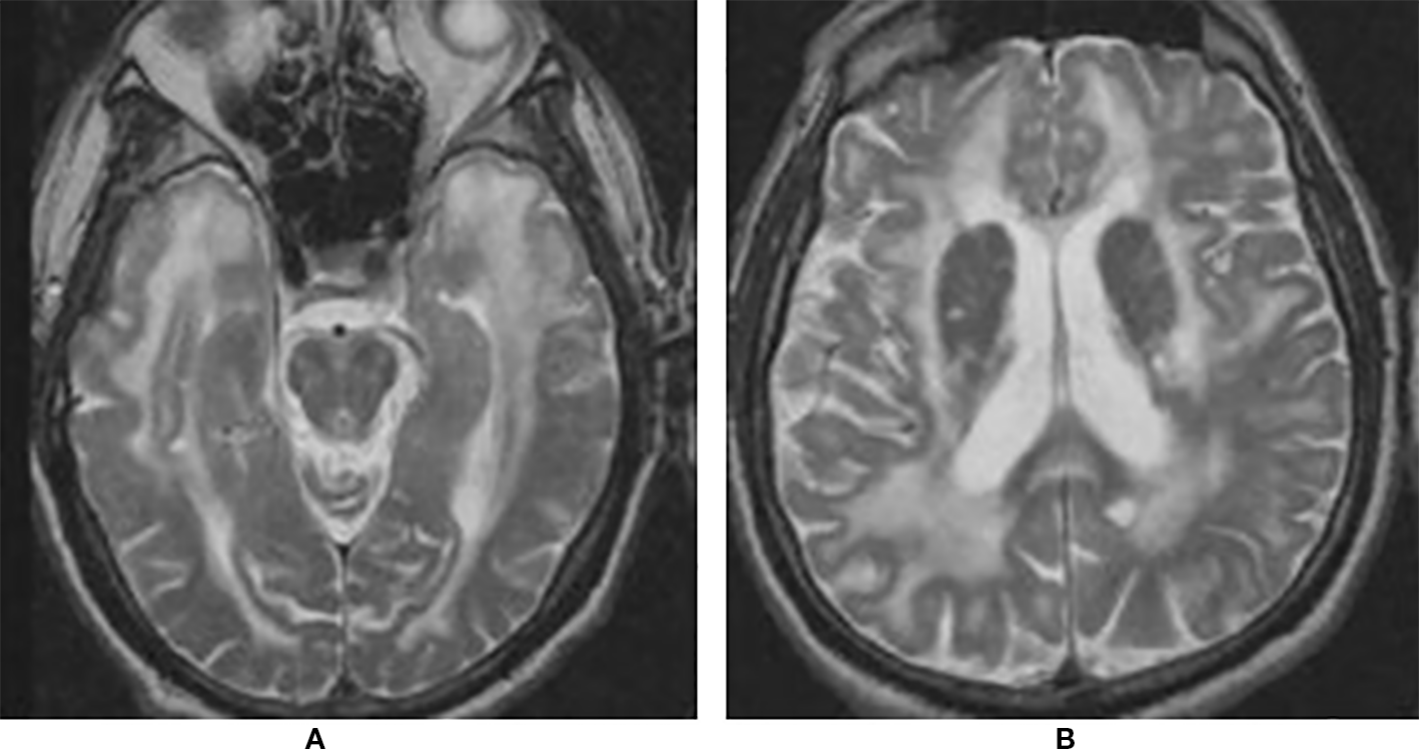
Brain magnetic resonance imaging (MRI) T2 showing extensive leukoencephalopathy with marked involvement of anterior temporal lobes **(A)** and external capsule **(B)**.

Recurrent cerebral ischemic events in a middle-aged adult with family history of strokes or dementia, especially if associated with migraine, mood or cognitive disorders, should raise the suspicion of CADASIL. Brain Magnetic Resonance Imaging (MRI) is the most useful imaging method to address the diagnosis. White-matters hyperintensities on T2-weighted or fluid-attenuated inversion recovery (FLAIR) sequences are the earliest and most frequent alterations, present in almost 90% of patients. Notably, the involvement of temporal poles is highly suggestive of CADASIL (Auer et al., 2001; Markus et al., 2001; O'Sullivan et al., 2001). Although white-matters hyperintensities are generally considered the consequence of hypoperfusion, recent evidence suggested a more peculiar underlying mechanism. In their study, De Guio and coworkers proposed that alterations of the temporal pole are the end result of an impairment of fluid drainage from the white matter, with consequent edema and variation in tissue water content (De Guio et al., 2018). Subcortical lacunar infarcts appear as punctiform areas of decreased signal at the gray-white matter junction and are usually detectable later in life (Chabriat et al., 1998; Herve et al., 2009). Other typical MRI findings include dilated perivascular spaces, especially in the basal ganglia, microhemorrages (also known as microbleeds), detected on gradient echo sequences in 31–69% of patients, and brain atrophy (Lesnik Oberstein et al., 2001; Dichgans et al., 2002; Cumurciuc et al., 2006; Peters et al., 2006; Jouvent et al., 2007) (**Figure 1**).

Although these MRI signs are, taken together, highly suggestive of CADASIL, demonstration of *NOTCH3* causative mutation at molecular analysis or characteristic ultrastructural abnormalities at skin biopsy are necessary to establish a diagnosis.

Genetic analysis is the gold standard, with 100% specificity and almost 100% sensitivity. Missense mutations are found in approximately 95% of patients, but in up to 5% patients genetic screening fails to give a diagnosis (Peters et al., 2005). Skin ultrastructural examination should be restricted to patients with negative molecular testing but a high level of suspicion based on clinical evaluation and brain imaging, or when a gene variant of unknown significance is detected (Chabriat et al., 2009). Ultrastructural analysis encompasses electron-microscopic and immunostaining techniques. Typical electron-microscopic findings consist of non-amyloid granular osmiophilic materials (GOMs) within the media of the skin arteries, while immunostaining is based on the use of *NOTCH3* protein-targeted monoclonal antibodies to detect the accumulation of *NOTCH3* protein in the vessel wall. While immunostaining is considered both sensitive (85–95%) and specific (95–100%), electron-microscopic testing has variable sensitivity and specificity (Joutel et al., 2001; Lesnik Oberstein, S. A. et al., 2003; Markus et al., 2001). However, the presence of GOMs is the pathological hallmark of CADASIL, being one of the criteria for the diagnosis, therefore its specificity can be considered 100%, while its sensitivity varies between operators (Mizuta et al., 2017).

### Pathogenesis

#### *NOTCH3* Properties

**Figure 2:**
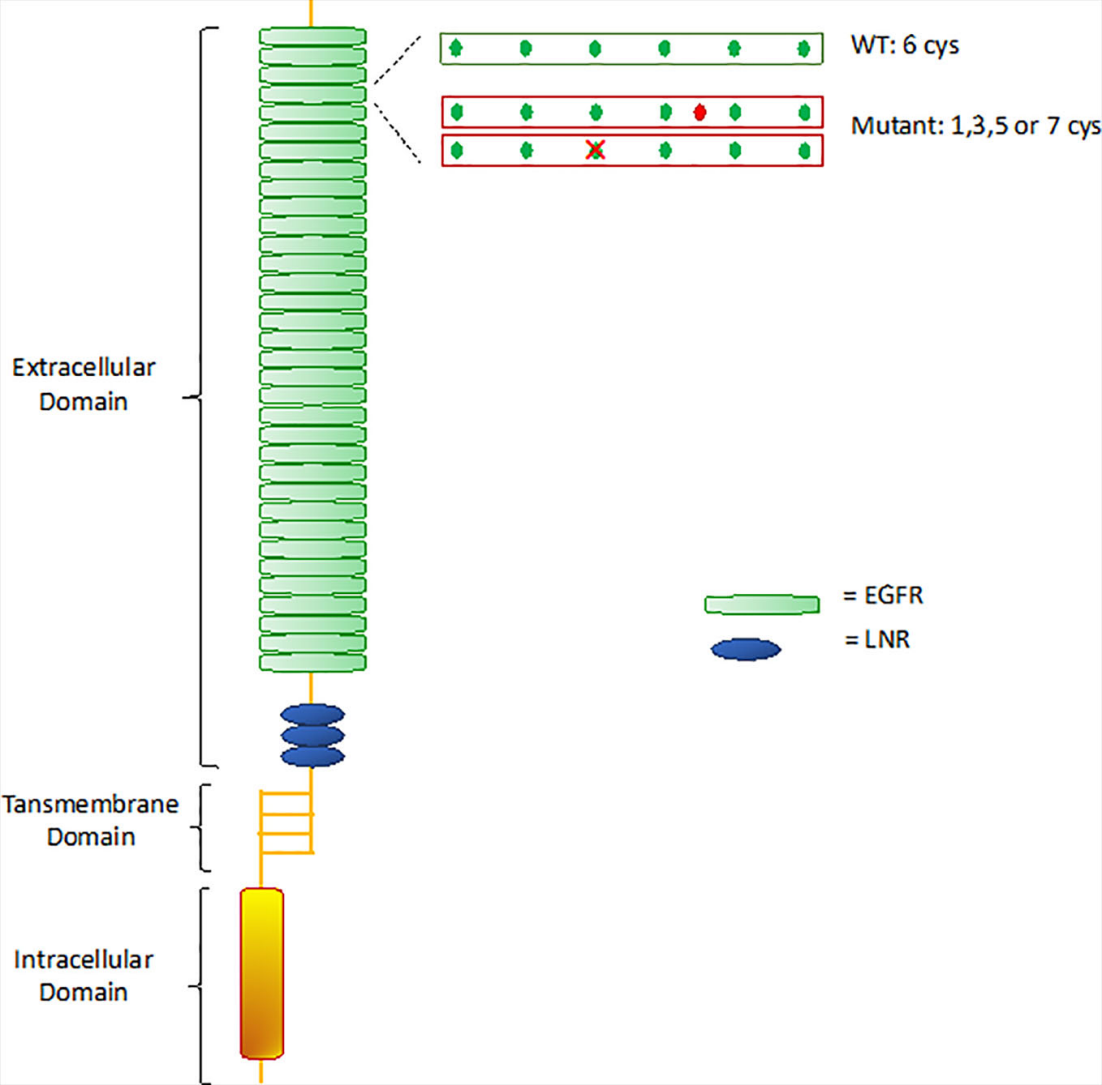
Notch 3 receptor structure. WT, wild type; EGFR, epidermal growth factor-like repeats; LNR, Lin12 repeats.

**Figure 3:**
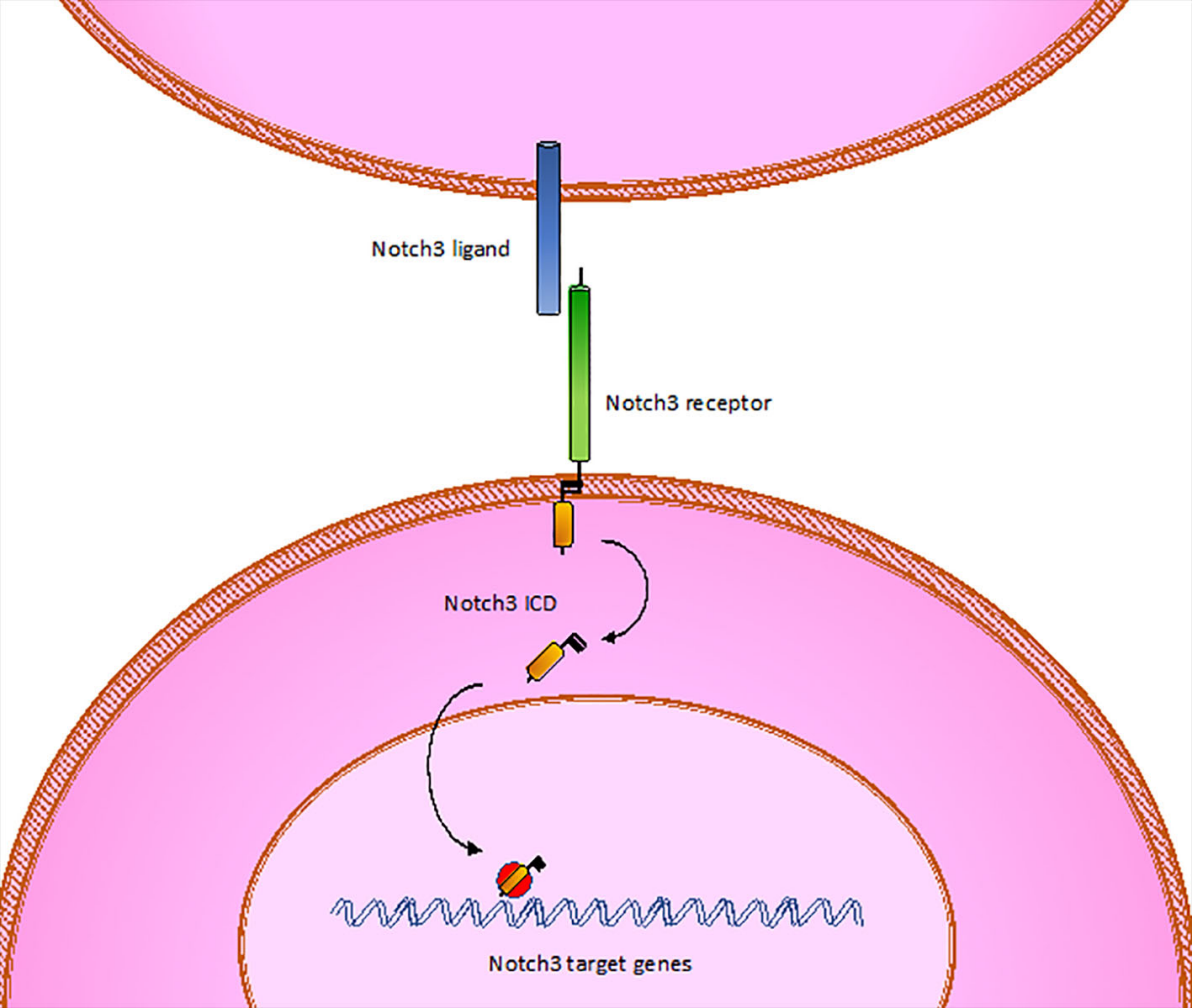
Wild type Notch3. ICD, intracellular domain.

CADASIL is caused by pathogenic mutations in *NOTCH3* gene on chromosome 19p13, transmitted in families in an autosomal dominant manner with variable penetrance. *NOTCH3* encodes a large transmembrane receptor, mainly expressed on vascular smooth muscle cells (VSMCs) of blood vessels and pericytes, composed of three domains: one extracellular domain (ECD), responsible for ligand-binding, one transmembrane domain, and one intracellular domain (ICD), responsible for intracellular signaling transduction (**Figure 2**). In *NOTCH3* signaling pathway, ligand binding induces a proteolytic cleavage of the ECD, which is released into the interstitial space between cells. This process makes the ICD to translocate to the nucleus and regulate the expression of specific target genes, involved in VSMCs differentiation, vascular development during embryogenesis, and vascular response to injury (Wang et al., 2002; Domenga et al., 2004; Andersson and Lendahl, 2014; Ghosh et al., 2015; Ferrante et al., 2019) (**Figure 3**). The ECD is composed of 3 Notch/Lin repeats and 34 epidermal growth factor-like repeats (EGFR) rich in cysteine residues which form disulfide bonds responsible for the protein's tertiary structure (Muiño et al., 2017).

Remarkably, almost all mutations in CADASIL involve one of the EGFR-encoding sequences. Mutations lead to changes in the number of cysteine residues, causing disulfide bridges disruption and receptor misfolding (Ishiko et al., 2006). Misfolded proteins are usually sequestered by the endoplasmic reticulum (ER) for refolding and trafficking, before ultimately being eliminated by the ER-associated degradation (ERAD) system (Ellgaard and Helenius, 2003; Calloni et al., 2005). Takahashi et al. (2010) demonstrated that mutant *NOTCH3* proteins are highly resistant to degradation, showing extremely slow turnover rates compared to wild-type (half-lives of 6 days for mutant vs 0.7 days for wild-type), thereby being retained in the endoplasmic reticulum (ER) for long time. Various studies suggested that retention of mutant aggregates causes ER stress with formation of reactive oxygen species (ROS), that obstacle cells growth and proliferation and prevent impaired cells replacement (Ihalainen et al., 2007; Takahashi et al., 2010). The biological mechanisms hindering mutant proteins degradation are still poorly understood. It is possible that misfolded proteins do not interact properly with chaperones and proteases that normally mediate proteins degradation. In particular, the chaperon calnexin was seen to co-localize with *NOTCH3* aggregates, suggesting that an abnormal prolonged association between *NOTCH3* aggregates and calnexin may facilitate retention of the aggregates in the ER and interfere with degradation by the ERAD pathway. Therefore, the specific interaction of mutant *NOTCH3* with calnexin could play a key role in CADASIL pathogenesis (Takahashi et al., 2010).

Cell lines expressing mutant *NOTCH3* are also more sensitive to stress inducers such as hypoxia, oxidative stress and glucose deprivation. These stressors induce proteasome dysfunction, activation of apoptotic pathways and cell death (Takahashi et al., 2010).

Interestingly, Viitanen et al. found out that mutant VMSCs had lower proliferation rates, but no higher apoptosis susceptibility, compared to healthy controls, suggesting that the mechanism responsible for thinner muscular layer of affected vessels may be the reduced proliferation of VSMCs rather than cell death (Viitanen et al., 2013). Moreover, they observed impaired mitochondria functioning in CADASIL VSMCs, with mutant cells showing increased irregular and abnormal mitochondria with altered membrane potential. This suggests a possible role of mitochondria in CADASIL pathology (Viitanen et al., 2013).

Some CADASIL mutations impair the activity of the *NOTCH3* receptor (Joutel et al., 2004). In particular, Joutel et al. found that the EGFR 10 and 11 are necessary for Delta/Jagged ligand binding, therefore mutations in those sites, detected in a minority of CADASIL families, may alter the receptor physiological functioning. These findings gave rise to the hypothesis that cells degeneration could be secondary to an impaired signaling activity, suggesting a *loss-of-function* mechanism at the bases of CADASIL pathogenesis (Joutel et al., 2004; Peters et al., 2004; Haritunians et al., 2005). However, more recent studies on transgenic mice revealed that the majority of CADASIL-associated mutations, including those located in EGFR 2-5, do not impair *NOTCH3* activity and that *NOTCH3* remains functional even when *NOTCH3*-ECD accumulates. Moreover, total loss of *NOTCH3* did not result in CADASIL pathology (Joutel, 2011). In this study, both EGFR10 and EGR2 mutated-*NOTCH3* maintained the ability to form *NOTCH3*-ECD aggregates, suggesting that *NOTCH3-*ECD accumulation is unrelated to signaling activity of the receptor (Joutel et al., unpublished). A genotype-phenotype correlation analysis revealed that EGFR10-11 and EGFR2-5 mutations are associated with different phenotypes, with EGFR10–11 mutants usually presenting with milder cognitive deficits, lower volume of lacunar infarcts, and higher volume of white matter hyperintensities at MRI, suggesting different pathological processes related to different *NOTCH3* mutations (Joutel, 2011). Altogether, these data suggest that compromised *NOTCH3* function may not be the primary determinant of CADASIL disease, prompting to speculation that CADASIL may be related to a protein gain-of-toxic function rather than loss of function (Monet et al., 2007; Joutel, 2011). Moreover, ECD-*NOTCH3* aggregates and GOM deposits are detected at a very early stage of the disease both in patients and mouse models, validating the hypothesis of toxic-proteins accumulation as the main cause of the disease (Joutel, 2011).

In conclusion, even if the possibility that loss of *NOTCH3* function may contribute to CADASIL pathogenesis cannot be totally excluded, the pathogenic effects of mutant *NOTCH3* is likely caused by its aggregate-prone property and resistance to degradation, which impair normal cells functioning and result in cells degeneration (Yamamoto et al., 2011). Interestingly, recent studies showed that not only VSMCs and pericytes are affected in CADASIL, but also endothelial cells and astrocytes, suggesting an overall impairment of the gliovascular unit (Pescini et al., 2017; Hase et al., 2018).

#### Molecular Findings

*NOTCH3* consists of 33 exons, but approximately 98% of CADASIL mutations occur in exons 2–23, which encode the 34 EGFR on the ECD. Over 40% of mutations are found in exons 3 and 4, which encode EGFR 2–5, with the majority of mutations involving exon 4. The prevalence of other variants shows a geographically-specific distribution, probably due to founder-mutations. For example, exon 3 is the second most frequent mutated exon in France, Germany, and England, as oppose to exon 11 in Dutch population (Joutel et al., 1997; Joutel et al., 2000; Markus et al., 2001; Peters et al., 2005; Lesnik Oberstein, S. A. et al., 2003). Mutations are mainly missense variants (95%), and less frequently small in-frame deletions, duplications, or splice-site mutations (Dichgans et al., 2000; Joutel et al., 2000; Dichgans et al., 2001; Dotti et al., 2004; Peters et al., 2005). The novo mutations have been also reported, but their frequency is unknown (Joutel et al., 2000; Coto et al., 2006). To date, more than 200 pathogenic variants have been described in CADASIL patients worldwide, all of them involving cysteine residues. Genetic variants not involving cysteine have also been described, but their pathogenic role in disease occurrence remains uncertain (Rutten et al., 2014; Rutten et al., 2019). Rutten et al. found a genotype-phenotype correlation depending on mutation position along the 34 EGFR domains. According to the results of these studies, individuals with EGFR 1–6 variants show a 12-year-earlier age at stroke onset, higher brain MRI lesions and lower survival rates compared to patients with EGFR 7–34 variants. Therefore, mutations in exons 7–34 may be associated with milder or later-onset CADASIL phenotype compared to EGFR 1–6 variants (Rutten et al., 2019).

However, no association was found between mutation site and clinical phenotype (Adib-Samii et al., 2010). Other cardiovascular risk factors, like hypertension and smoking, have been demonstrated to contribute to disease severity. Hypertension increases the risk of acute stroke in CADASIL as in sporadic small-vessel disease. Smoking was associated with an earlier onset of stroke in CADASIL patients, suggesting that it may facilitate acute occlusion of perforating arteries. On the contrary, the association between stroke and body mass index, raised cholesterol, or elevated serum homocysteine was not demonstrated in CADASIL (Adib-Samii et al., 2010). Interestingly, a recent study found significant taxonomic differences in gut microbiota between CADASIL patients and controls, suggesting a possible role of gut microbiota in influencing disease onset and progression, but more research is needed to confirm this hypothesis (Matsuura et al., 2019). The role of cardiovascular risk factors could be particularly important in EGFR 7–34 mutated individuals, who needs additional risk factors for the classical CADASIL phenotype to develop, while mutations in EGFR 1–6 may have a strong enough effect to cause the disease. These observations completely change the classic CADASIL-*NOTCH3* disease paradigm, and lead to speculate *NOTCH3* disease as a spectrum of conditions which differ in age of onset, severity, and association with external risk factors. Investigating patients at risk of more severe disease is likely to facilitate the identification of disease-specific biomarkers and eventually lead to powerful strategies for disease prevention and therapeutic intervention (Rutten et al., 2019).

#### Histopathological Findings

Although CADASIL is a systemic microangiopathy, vascular complications are largely limited to the brain, in particular to the small penetrating vessels and leptomeningeal arteries (Miao et al., 2004). Microscopic analysis of affected vessels shows atrophic myocytes, that can eventually disappear with disease progression. VSMCs degeneration results in a gradual but relentless degradation of the muscular layer in cerebral blood vessels, with an eventual breakdown in the integrity of the vascular wall (Chabriat et al., 2009). Over time, VSMCs apoptosis leads to fibrosis and thickening of the arterial wall, progressive lumen stenosis and vascular insufficiency that makes the already poorly perfused terminal regions particularly susceptible to infarcts. SMCs degeneration is followed by the emergence of large, rounded, bloated cells, named balloon cells, predominantly in the border between the degenerating medial layer and the adventitia. A recent study indicated that these cells express mRNAs for vascular smooth muscle genes and could be therefore considered of vascular smooth muscle origin (Gatti et al., 2019). Their presence in the degenerating medial layer is probably the marker of smooth-muscle cells degeneration (Geschwind et al., 2018). Wall thickening of leptomeningeal and deep penetrating arteries of the white matter, a histopathological hallmark of CADASIL, is predominantly a result of marked intimal hyperplasia (Dong et al., 2013). Surprisingly, a recent study focusing on the maturation of cerebral intimal cells in CADASIL observed that the intima contains cells that express mature smooth muscle markers (Gatti et al., 2019). One possible explanation is that those neointimal cells are the result of medial SMCs trans-differentiation and migration in response to vascular injury, a pathway of injury-response already described in peripheral vessels (Yang et al., 2016; Gatti et al., 2019). An alternative hypothesis is that that neointimal SMCs originate from circulating pluripotent cells, which burrow into the intimal layer where they are exposed to biochemical factors which induce differentiation into SMCs (Tang et al., 2012; Gatti et al., 2019).

Progressive vascular thickening can eventually result in lumen occlusion, with ultimate subcortical lacunar infarcts and cribriform alterations (Viswanathan et al., 2006). However, some studies suggested that the thickening of the wall may be not directly associated to luminal narrowing (Liem et al., 2010b; Dong et al., 2013). A 7-T MR angiography study showed no differences in luminal caliber of lenticulostriate arteries between CADASIL and control subjects (Liem et al., 2010b). This evidenced was confirmed by a pathological study on CADASIL brain samples, which reported a thickening of leptomeningeal arteries, especially secondary to intimal hyperplasia, that did not affect lumen diameter (Dong et al., 2013). Therefore, hypoperfusion in CADASIL may be not directly attributable to arterial narrowing, but maybe to other hemodynamic flow alterations like impaired cerebrovascular reactivity (Liem et al., 2010b).

Chronic global cerebral hypoperfusion decreases neuronal threshold to cortical spreading depression (CDS) and depolarization, thus facilitating migraine with aura occurrence (Liem et al., 2010a). Moreover, blood flow reduction in frontal-temporal white matter results in a hypoperfusion-mediated neuronal degeneration with progressive cortical atrophy, accounting for cognitive impairment in CADASIL. Previous studies have, actually, demonstrated that cerebral blood flow is reduced in demented CADASIL patients compared to patients with no cognitive impairment (Carare et al., 2013).

Several MRI and histopathological studies revealed cerebral microbleeds scattered throughout the brain of most CADASIL patients. Microbleeds were found within intact brain areas, without a clear prevalence of specific structures. These findings indicate that hemorrhages occur independently from ischemic lesions, suggesting that CADASIL microangiopathy is the underlying cause of both ischemic lesions and petechial hemorrhages. The clinical implication of microbleeds is still unknown. In an Italian-British cohort study, microbleeds were detected in in 34% of CADASIL patients, and their number was positively correlated with age. The presence of microbleeds, in particular in lobar regions, was significantly associated with hemorrhagic stroke, dementia, and urge incontinence, while infratentorial and deep microbleeds were associated with dementia and urge incontinence (Nannucci et al., 2018). Interestingly, a recent large cohort study reported a significant association between microbleeds and the risk of ischemic stroke in CADASIL, patients with microbleeds having a 2-fold increased risk of ischemic stroke compared to patients without microbleeds, independently of age, history of stroke, sex, vascular risk factors, and use of antiplatelet agents (Puy et al., 2017). The presence of microbleeds could, therefore, identify a subgroup of patients with severe forms of the disease, at risk both of ischemic and hemorrhagic lesions, and not only quantify the risk of intracranial bleeding (Puy et al., 2017).

As mentioned before, non-amyloid GOMs within the media of the affected vessels is a typical finding in affected individuals. GOMs appear as 0.2–2 mm-sized osmiophilic and periodic-acid Schiff-positive granules located around degenerating VSMCs. GOMs molecular composition is still under investigation. Recent studies revealed mutant ECD of *NOTCH3*-encoded receptor and of other matrix proteins involved in blood vessel maintenance, such as tissue inhibitor of metalloproteinases 3 (TIMP-3), vitronectin (VTN), and latent TGF-β binding protein 1 (LTBP-1) (Carare et al., 2013; Monet-Lepreˆtre et al., 2013; Yamamoto et al., 2013; Kast et al., 2014), as well as amyloid P, annexin 2 and periostin (Nagatoshi et al., 2017). Amyloid P, which has been extensively investigated in Alzheimer disease because of its role in the stabilization of amyloid plaques, may exert an analogous role in the stabilization of GOMs in CADASIL (Nagatoshi et al., 2017). The processes that lead to GOMs formation are far from being understood. It has been hypothesized that GOMs are formed on the cell surface, where mutant *NOTCH3* ECD may acquire the ability to recruit extracellular proteins to create large aggregates. It is also possible that GOMs are assembled within the ER, where mutant *NOTCH3* aggregates are retained, and are released in the extracellular space after VSMCs apoptosis (Takahashi et al., 2010).

Electron microscopy analysis on skin, skeletal muscle, kidney, and pericardial samples of CADASIL patients identified GOMs not only around VSMCs and pericytes of the brain, but also in the above-mentioned tissues, supporting the idea that CADASIL is a systemic disease and not only a disorder confined to the CNS (Lewandowska et al., 2010; Morroni et al., 2013). Moreover, Lorenzi et al. found that several GOM deposits were surrounded by an electron-lucent halo, probably made of more densely packed extracellular matrix around the GOM which interferes with ubiquination and transendocytosis of mutant NOTCH3, preventing GOM clearance and promoting protein accumulation (Lorenzi et al., 2017). Therefore, the halo around GOMs may represent the morphological evidence of aberrant NOTCH3 processing, and could be considered as an ultrastructural marker for CADASIL (Lorenzi et al., 2017). However, GOM formation and development over time and their role in disease progression remain largely unknown. A recent study found that GOM deposits are not static, but rather change in terms of size, morphology, and number during disease course in CADASIL transgenic mice (Gravesteijn et al., 2019). The authors found a progression from initially small round GOMs, located to the abluminal side of mural cells, to large amorphous deposits which induced basement membrane bulging and mural cell indentation, seen in the last stages of the disease, and proposed a GOM classification system divided into five stages of progression. Notably, both in aged mice and in post-mortem CADASIL brain microvessels, large and amorphic GOM deposits coexisted with small and recent ones, suggesting that GOMs are continuously formed during disease progression (Gravesteijn et al., 2019).

GOM deposits adhere to microvascular walls and accumulate in perivascular drainage routes, obstructing perivascular drainage of fluid and solutes. Combined with the reduced vascular tone of arterioles and the consequent weakening of pulsations for lymphatic drainage, the result is further accumulation of proteins around the blood vessels (Weller et al., 2009). This explains the typical enlargement of perivascular spaces observed in CADASIL patients (Chabriat et al., 2009). Not only VSMCs, but also pericytes, endothelial cells and astrocytes are primarily affected in CADASIL. Pericytes degeneration causes a progressive loss of astrocytic end-foot processes contact with capillaries that lead to disruption of the blood-brain barrier, resulting in increased permeability to neuro-toxic substances that can contribute to neuronal damage (Ghosh et al., 2015). This progressive loss of neurons is the consequence of both vascular insufficiency and blood-brain barrier disruption.

### Therapeutic Approach

#### Novel Therapeutic Approaches

CADASIL is a progressive and fatal disease, for which no disease-modifying treatment has been made available to date. However, the discovery that the disease is caused by *NOTCH3* cysteine-altering mutations has led to several promising experimental approaches.

Recently, Rutten et al. proposed a novel therapeutic strategy based on cysteine corrective exon skipping (Rutten et al., 2016). In their study, the investigators transfected patient-derived VSMCs with Antisense Oligo Nucleotides (AON) targeted to specific mutated *NOTCH3* exons to alter pre-mRNA expression, thus preventing the inclusion of mutated exons in mature mRNA. This resulted in *NOTCH3* skip proteins in which the unpaired cysteines were eliminated and disulfide bridges correctly formed. The novel skip proteins were correctly localized on the cell surface, as in wild-type *NOTCH3,* and maintained ligand binding capacity and proper ligand induced signaling. Moreover, after transfection, total *NOTCH3* expression levels were maintained and *NOTCH3* downstream target genes were correctly expressed. An alternative AON-based approach was targeted to induce NOTCH3 downregulation. The idea was to use AONs that induced RNase-mediated degradation of RNA, thus leading to a reduction of mutant *NOTCH3* protein expression and therefore, theoretically, to a reduction of *NOTCH3* aggregates deposition. After transfection of VSMCs with downregulating *NOTCH3* gapmer AONs, quantitative PCR analysis showed an effective reduction of *NOTCH3* expression levels and reduced expression of NOTCH3 target genes. Although these AON-based approaches could represent a therapeutic strategy for CADASIL, *in vivo* studies are needed to confirm these promising *in vitro* findings. In fact, cell-models do not reproduce CADASIL hallmarks like *NOTCH3*-ECD aggregation, GOM formation and VSMC degeneration, which play an important role in disease pathogenesis and therefore represent cardinal target to address in order to effectively contrast the disease. In that respect, creation of transgenic mouse models with typical CADASIL features is ongoing, so that *NOTCH3* exon skipping strategy and AON-mediated downregulation will be tested *in vivo* to determine whether this approach influences *NOTCH3* aggregation and improves clinical outcome (Rutten et al., 2016).

Another therapeutic attempt was done with immunotherapy. Ghezali et al. generated a monoclonal antibody (5E1) targeting the ECD of *NOTCH3* protein, aimed to reduce NOTCH3 aggregates and GOM deposition. This hypothesis raised from the evidence that, in mouse models of Alzheimer disease, administration of antibodies against amyloid β peptides leads to a reduction of extracellular β amyloid plaques (Golde et al., 2009). One of the mechanisms proposed was that antibody binding to amyloid plaques could activate the surrounding microglia/macrophages with consequent phagocytosis of existing plaques. In that study, intraperitoneal injections of 5E1 in transgenic mice showed that 5E1 clone could detect *NOTCH3*-ECD deposits, but failed to reduce *NOTCH3*-ECD/GOM deposition. However, 5E1 injection had beneficial effects on cerebrovascular function by normalizing vasodilatory responses and myogenic tone (Ghezali et al., 2018). The evidence that peripheral immunotherapy targeting *NOTCH3*-ECD protects against cerebrovascular dysfunction despite continued *NOTCH3*-ECD accumulation/GOM deposition might challenge the concept of proteins accumulation as the driving force in CADASIL, emphasizing the role of other possible mechanism, such as cerebrovascular flow hemodynamics, in the pathogenesis of the disease (Ghezali et al., 2018). More studies are required to determine if this approach could have beneficial effects in CADASIL patients (Ghezali et al., 2018).

Subcutaneous administration of stem cell factor (SCF) in combination with granulocyte colony-stimulating factor (G-CSF) in a mouse model of CADASIL showed to improve cognitive function, inhibited VSMCs apoptosis and cerebral capillary damage, increased cerebral blood vessel density and promoted neurogenesis (Liu et al., 2015). In CADASIL patients, blood levels of endothelial progenitor cells (EPCs) are significantly lower than in control subjects (Pescini et al., 2010), probably due to their persistent consumption as an attempt to replace the damaged endothelium. This evidence could explain, at least in part, the beneficial effects of SCF + G-CSF administration in restricting the pathological progression of CADASIL. Moreover, although the mechanisms responsible for the SCF + G-CSF-mediated beneficial effects remain largely unknown, it has been hypotized that SCF + G-CSF may act on caspase-3 pathway, thus inhibiting VSMC apoptosis (Liu et al., 2015). Two clinical trials testing the effects of Bone Marrow Derived Stem Cells (BMSCs) administration in patients with various neurological disorders, including CADASIL, are actually ongoing (ClinicalTrials.gov).

#### Current Therapy and Prevention

At present, patients diagnosed with CADASIL only receive symptomatic treatments, mainly based on regular clinical practice. Acute stroke care in CADASIL consists of intravenous tissue plasminogen activator (tPA) if criteria are met, while endovascular recanalization is unlikely to be useful, since CADASIL is a small vessel disease (Khan et al., 2016). Secondary prevention includes all the common risk reduction strategies like limited alcohol consumption, low blood cholesterol levels, weight control, regular physical activity, and balanced diet. Smoking cessation may be particularly important, since smoking is associated with an earlier age of onset and increased risk of stroke and migraine (Singhal et al., 2004; Adib-Samii et al., 2010). Hypertension seems to facilitate micro hemorrhages and to accelerate disease progression (Viswanathan et al., 2006). However, low blood pressure is associated with increased incidence of dementia in CADASIL patients, therefore care should be taken to avoid excessive pressure lowering (Guo et al., 2001; Verghese et al., 2003). In that respect, a European clinical trial studying the effects of amlodipine and other blood pressure lowering agents on microvascular function in small vessel diseases is actually ongoing (ClinicalTrials.gov). Diabetes control is also essential, since Hemoglobin A1c (HbA1c) is correlated with cerebral micro-hemorrhages (Viswanathan et al., 2006; Ho et al., 2011). Hyper-homocysteinemia is a novel proposed risk factor for stroke and cerebral small vessel disease, even if homocysteine-lowering therapy in secondary stroke prevention failed to show any benefit (Ho et al., 2011; Martí-Carvajal et al., 2015). However, homocysteine-lowering therapy with B vitamins may be useful in CADASIL patients with high homocysteine levels who are not on antiplatelet medications. (Hassan et al., 2004). General recommendations support low dose aspirin alone or in combination with clopidogrel in secondary stroke prevention, but the specific benefit of antiplatelet therapy in CADASIL is still to be assessed (Maclean et al., 2005; Bersano et al., 2017). To date, no data support the use of oral anticoagulants (Lesnik Oberstein et al., 2001).

Statins are the treatment of choice if hypercholesterolemia is present, but atorvastatin did not show to improve vascular reactivity or to affect mean flow velocity in CADASIL patients (Peters et al., 2007). Two studies described a beneficial effect of acetazolamide on cerebral perfusion, and lomerizine was reported to raise cerebral blood flow in one case report, but larger studies are required to properly define their clinical benefits (Chabriat et al., 2003; Mizuno et al., 2009; Huang et al., 2010).

Acute migraine attacks should be treated with common analgesics such as paracetamol, acetaminophen-aspirin-caffeine fixed combination and antiemetics (Goldstein et al., 2014). Evidences suggest that triptans and ergot derivatives should be use with caution due to their vasoconstrictive effect and their harmful action on capillary endothelium (Tfelt-Hansen et al., 2000; Ferrari et al., 2001). Prophylactic migraine strategies include good lifestyle and drugs normally used for migraine patients, with caution for amitriptyline, beta-blockers, flunarizine, and topiramate since they may worsen mood and cognitive symptoms (Finocchi et al., 2010). One study demonstrated a beneficial effect of acetazolamide on migraine (Donnini et al., 2012). CADASIL patients with migraine seem to have higher blood homocysteine levels, and B vitamin supplementation lowering homocysteine was seen to decrease migraine severity and frequency (Bousser and Tournier-Lasserve, 2001).

No drugs have clearly shown benefit on cognitive functions. A randomized-controlled trial on donepezil failed to show an improvement of cognition in CADASIL patients compared to controls, even if the donepezil group showed better executive functions (Schneider, 2008). Treatment with acetylcholinesterase inhibitor galantamine showed some benefit on 4 CADASIL patients in terms of behavior and caregiver burden, suggesting the existence of a cholinergic deficit in CADASIL, but a larger trial is required to confirm this hypothesis (Posada et al., 2008). Lastly, L-dopa administration, which has been seen to improve cholinergic activity in Alzheimer's patients, had no effects in CADASIL, supporting the theory that acetylcholine and dopamine systems impairment is not relevant in this condition (Nardone et al., 2014).

Psychiatric symptoms treatment in CADASIL should follow the best general clinical practice. A recent meta-analysis found an association between selective serotonin reuptake inhibitors (SSRI) and increased risk of ischemic and hemorrhagic stroke (Shin et al., 2017), but more researches are needed before discouraging the administration of these drugs, which are widely used for the treatment of mood disorders in small vessel disease and stroke patients (Mead et al., 2012). Quietiapine, risperidone, sodium valproate, and flupentixol showed clinical benefits in some case reports (Park et al., 2014; Ho and Mondry, 2015).

### CADASIL as a Model of Sporadic Small-Vessel Disease

Besides being the most common familiar form of SVD, CADASIL may represent a model to explore the pathogenesis of sporadic SVD (Joutel et al., 1996). Like CADASIL, sporadic SVD usually manifests with multiple lacunar strokes, mostly ischemic, sometimes leading to vascular cognitive impairment and vascular dementia (Pantoni, 2014). It also shares a number of radiological features with CADASIL, including white-matters hyperintensities on MRI T2/FLAIR sequences, cerebral microbleeds, ampliated perivascular spaces, and brain atrophy (Wardlaw et al., 2013). Furthermore, histopathological features typical of CADASIL including fibrosis of the adventitia, smooth muscle cells necrosis and loss of the endothelial cells of the perforating arteries resemble those seen in sporadic disease (Arima et al., 2003). Therefore, specific pathogenic pathways may underline the two conditions and contribute to both the sporadic and the genetic forms of SVD. Pathognomic of CADASIL is the presence of GOM deposits in perivascular spaces, which promote matrisome protein sequestration thus impairing extracellular matrix functioning (Monet-Lepreˆtre et al., 2013). Interestingly, perturbations of the cerebrovascular matrisome are emerging as key factors also in determining sporadic SVD (Joutel et al., 2016). Moreover, increased blood brain barrier permeability, seen in CADASIL as a consequence of pericytes damage, seems to be present also is sporadic SVD (Wardlaw et al., 2003). Population-based genetic studies also hint the possible involvement of *NOTCH3* gene in sporadic SVD. In an Austrian study, four common single nucleotide polymorphisms (SNPs) in the *NOTCH3* gene region were found to be associated with the presence of white-matter hyperintensities in hypertensive subjects, suggesting that minor alterations in *NOTCH3* receptor may act in combination with hypertension to determine white matter abnormalities (Schmidt et al., 2011). These evidences support the prevailing idea of a convergence of pathways between genetic and sporadic forms of SVD. The main difference between the two conditions is that CADASIL, other than being genetically determined, usually manifests at early age while sporadic SVD is an age-dependent disease in which multiple aging-related cardiovascular and cerebrovascular risk factors act together to determine the pathology of small arteries (Charidimou et al., 2016). This makes CADASIL an attractive model of sporadic cerebral SVD in which the influence of external confounders, including aging and aging-related risk factors, has been removed. Therefore, investigating CADASIL molecular alterations may be crucial in understanding the mechanisms underlying sporadic SVD, depicting CADASIL as an appropriate model to study sporadic SVD (Tan et al., 2017).

## Conclusion

CADASIL is the most important cause of inherited stroke and vascular cognitive impairment in middle-aged adults. To date, the exact mechanisms underlying the disease are far from being understood. However, the discovery of *NOTCH3* mutations as the cause of the disorder promoted several experimental studies that may provide new insights on CADASIL. Promising therapeutic approaches are under investigation which may lead in the following years to new treatment strategies. Since CADASIL can be conceptualized as a model to investigate sporadic SVD, continuous efforts aimed at understanding the pathogenesis of this monogenic disorder are necessary and should strongly be encouraged in order to deepen our knowledge and to develop novel treatment options against the degenerative process of cerebral small vessels.

## Author Contributions

ML drafted the manuscript. APa and APe reviewed and modified the manuscript.

## Conflict of Interest

The authors declare that the research was conducted in the absence of any commercial or financial relationships that could be construed as a potential conflict of interest.
